# Multifunctional Metal Polyphenol Nanoparticles Based on Photothermal Therapy for Bacterial Eradication and Wound Healing

**DOI:** 10.34133/bmr.0387

**Published:** 2026-09-01

**Authors:** Haitao Yuan, Mengyun Hou, Min Zou, Shiyan He, Yunmeng Bai, Wenzhe Chen, Junhui Chen, Xiaoxian Wang, Jingbo Ma, Jinyue He, Centing Wang, Xinmiao Liu, Lihong Jiang, Li Song, Wei Xiao, Jigang Wang

**Affiliations:** ^1^ Center for Drug Research and Development, Guangdong Provincial Key Laboratory for Research and Evaluation of Pharmaceutical Preparations, Guangdong Pharmaceutical University, Guangzhou 510006, P.R. China.; ^2^ Department of Clinical Laboratory, Guangdong Provincial Clinical Research Center for Geriatrics, Shenzhen Clinical Research Center for Geriatrics, Shenzhen People's Hospital (The Second Clinical Medical College, Jinan University; The First Affiliated Hospital, Southern University of Science and Technology), Shenzhen 518020, P.R. China.; ^3^ Guangdong Provincial Key Laboratory of New Drug Screening, School of Pharmaceutical Sciences, Southern Medical University, Guangzhou 510515, P.R. China.; ^4^ Center for Translational Research in Clinical Medicine, Medical School, Kunming University of Science and Technology, Kunming, P.R. China.; ^5^ State Key Laboratory for Quality Ensurance and Sustainable Use of Dao-di Herbs, Artemisinin Research Center, and Institute of Chinese Materia Medica, China Academy of Chinese Medical Sciences, Beijing 100700, P.R. China.; ^6^ Shandong Academy of Chinese Medicine, Jinan 250000, P.R. China.

## Abstract

Balancing short-term antibacterial needs with long-term anti-inflammatory effects remains a major challenge in wound healing. Multifunctional bioactive materials capable of both efficient antibacterial action and inflammation modulation represent a promising solution. However, the majority of traditional antibacterial biomaterials possess only a single antibacterial effect, and their synthesis and preparation are intricate, which might restrict their clinical transformation. Chlorogenic acid is a natural compound endowed with anti-inflammatory properties. However, it is beset by certain inherent drawbacks, including poor water solubility and limited bioavailability. To overcome these difficulties, we have fabricated multifunctional nanoparticles (chlorogenic acid–iron nanoparticles, CA-Fe NPs) through the co-assembly of chlorogenic acid and iron ions in a straightforward manner. We found that CA-Fe NPs exhibit excellent photothermal conversion performance in vitro. Upon near-infrared (NIR) irradiation, they exhibit potent broad-spectrum antimicrobial activity against *Staphylococcus aureus*, *Escherichia coli*, *Candida albicans*, *Klebsiella pneumoniae*, and *Pseudomonas aeruginosa*. The CA-Fe NPs markedly reduced H_2_O_2_-induced reactive oxygen species levels and apoptosis in epithelial cells and suppressed lipopolysaccharide-induced M1 macrophage polarization in RAW 264.7 cells. Transmission electron microscopy results revealed enhanced bacterial membrane disruption by CA-Fe NPs under NIR irradiation, causing pronounced protein leakage. Transcriptomic analysis indicates that CA-Fe NPs combined with NIR disrupt the tricarboxylic acid cycle, cell-wall organization, and other metabolic processes. In vivo, within a methicillin-resistant *Staphylococcus aureus*-infected skin wound model, CA-Fe NPs maintained photothermal efficacy, effectively reduced serum levels of interleukin-1 beta, interleukin-6, and tumor necrosis factor alpha, and accelerated wound healing. These findings suggest that CA-Fe NPs are multifunctional materials with broad-spectrum bactericidal ability, antioxidant, anti-inflammatory, and wound-healing-promotion properties. These nanoparticles possess promising prospects for biomedical applications.

## Introduction

Wound infections are mostly induced by bacterial colonization, which tends to cause local tissue infection. Without timely and effective intervention, the infection may develop into a chronic condition or even progress to severe systemic infection [1]. At present, antibiotics remain the first-line clinical treatment for wound infections [2]. However, long-term abuse and inappropriate use have directly led to the emergence and spread of bacterial resistance [3]. Bacterial resistance, the most concerning type of antimicrobial resistance, has become a major global public health crisis, imposing a heavy burden on socioeconomic development [4,5]. According to statistics in 2019, bacterial resistance directly caused 1.27 million deaths and was associated with an additional 4.95 million deaths [4]. Without effective control strategies, the annual death toll caused by drug-resistant bacterial infections is projected to rise to 10 million by 2050 [6]. Among these, *Escherichia coli*, *Staphylococcus aureus*, *Klebsiella pneumoniae*, *Streptococcus pneumoniae*, *Acinetobacter baumannii*, and *Pseudomonas aeruginosa* (known as ESKAPE pathogens) are the 6 major pathogens responsible for resistance-related deaths [4,7]. Therefore, developing novel antibacterial agents or non-antibiotic alternative strategies to combat bacterial resistance has become an urgent issue.

In recent years, the rapid development of nanotechnology has brought a new breakthrough in the antibacterial field. Numerous studies have confirmed that nanozymes exhibit excellent antibacterial activity due to their unique physicochemical properties [8–10]. Unlike traditional antibiotics that act by targeting specific proteins, nanozymes mainly catalyze the production of large amounts of reactive oxygen species (ROS), causing irreversible oxidative damage to bacterial cell membranes, genetic materials, and proteins [11–13], thereby achieving a broader spectrum of antibacterial effects [14].

Previous studies have shown that various metal and metal oxide nanomaterials (such as Cu_2_MoS_4_ nanoparticles [NPs] [9], gold–platinum nanodots [15], and CuO_2_ nanodots [16]) possess enzyme-like catalytic activity and can achieve efficient bacteriostasis and sterilization by regulating ROS levels. Meanwhile, these nanomaterials also have outstanding advantages, including low cost, high stability, and tunable catalytic activity [17]. To further improve antibacterial efficiency, researchers have focused on constructing multifunctional integrated nano-antibacterial systems. For example, the manganese-based metal-organic framework–graphene oxide–silver (Mn-MOF-GO-Ag) composite developed by Yin et al. [18] enables transdermal drug delivery and accelerates diabetic ulcer wound repair. The UiO-66-NH-CO-MoS_2_ nanocomposite prepared by Liao et al. [10] achieves efficient bacterial elimination by integrating photothermal therapy, photodynamic therapy, and peroxidase-like activity.

Natural products have become an important reservoir for the development of novel antibacterial molecules due to their wide sources, good biocompatibility, and high safety [19]. Chlorogenic acid is a polyphenolic natural product widely found in tea, coffee, honeysuckle, and other plants, which has been proven to exert various biological functions, including neuroprotection, anti-inflammation, antioxidant, and antibacterial effects [20,21]. Therefore, the construction of multifunctional nanomaterial antibacterial systems based on natural products provides a promising new strategy to address bacterial resistance.

In this study, chlorogenic acid–iron nanoparticles (CA-Fe NPs) with both photothermal performance and anti-inflammatory activity were prepared via the chelation of chlorogenic acid and iron ions. These NPs showed excellent photothermal conversion efficiency and exhibited high bactericidal efficacy against *S. aureus*, *E. coli*, *C. albicans*, *K. pneumoniae*, and *P. aeruginosa*, demonstrating favorable broad-spectrum antibacterial activity. Transcriptomic analysis revealed that CA-Fe NPs combined with near-infrared (NIR) irradiation markedly down-regulated key metabolic pathways in *S. aureus*, including the tricarboxylic acid cycle, antibiotic response, phosphorylation signal transduction, and cell-wall synthesis. In vitro experiments demonstrated that CA-Fe NPs effectively alleviated oxidative-stress-induced cell apoptosis and damage and potently inhibited lipopolysaccharide (LPS)-induced polarization of RAW 264.7 macrophages toward the pro-inflammatory M1 phenotype.

**Fig. 1.**
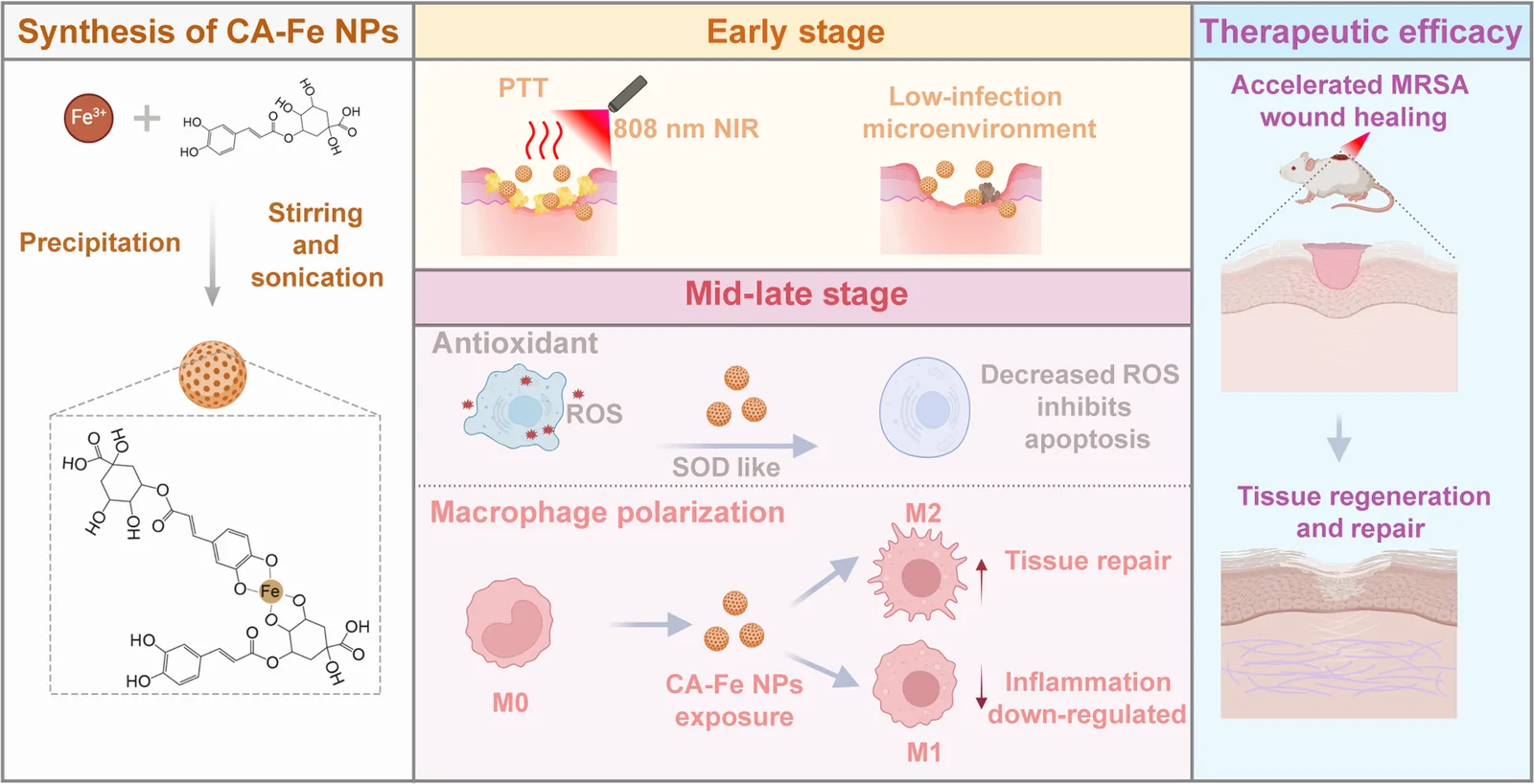
Schematic illustration of chlorogenic acid–iron nanoparticles (CA-Fe NPs) for the treatment of methicillin-resistant *Staphylococcus aureus* (MRSA)-infected wounds. PTT, photothermal therapy; NIR, near-infrared; ROS, reactive oxygen species; SOD, superoxide dismutase.

In a mouse model of methicillin-resistant *Staphylococcus aureus* (MRSA)-infected wounds, CA-Fe NPs markedly reduced local inflammation and promoted tissue repair and regeneration, showing great potential for the treatment of drug-resistant bacterial infections (Fig. 1).

## Materials and Methods

### Synthesis and characterization of CA-Fe NPs

Chlorogenic acid (10 mg) and FeCl_3_ (10 mg) were dissolved separately in absolute ethanol (2 ml) via sonication (5 min) and mixed. Ultrapure water (5 ml) was added dropwise under magnetic stirring, followed by overnight incubation. Ethanol was removed using a rotary evaporator, and the resulting CA-Fe NPs were stored at 4 °C in the dark. The hydrodynamic size and zeta potential of CA-Fe NPs (0.1 mg/ml) were determined using a Malvern Zetasizer Nano ZS90 instrument at 25 °C. Samples were dispersed in ultrapure water or 1× phosphate-buffered saline (PBS, pH 7.4) and measured in triplicate, with results expressed as mean ± standard deviation. UV–Vis and fluorescence spectra were recorded on JASCO V-550 and FP-6500 spectrometers, respectively. Morphology was characterized via high-resolution transmission electron microscopy (FEI TECNAI G2, 200 kV).

### SOD-like activity assay

The superoxide anion scavenging activity of chlorogenic acid or CA-Fe NPs (10, 50, 100, and 500 μg/ml) was determined using a total superoxide dismutase (SOD) activity assay kit. Absorbance at 450 nm was measured for blank, blank control, sample, and sample blank groups (denoted as AB, ABC, AT, and ATC). The scavenging rate was calculated as follows: scavenging rate (%) = [(AB − ABC) − (AT − ATC)] / (AB − ABC) × 100%. Experiments were performed in duplicate.

### Bacterial culture and in vitro antibacterial assay

*S. aureus*, *E. coli*, and *C. albicans* were cultured to mid-log phase (optical density at 600 nm [OD_600_] = 1.0) in Luria-Bertani (LB) broth at 37 °C with shaking (220 rpm). For antibacterial assays, bacteria were divided into 4 groups: (a) control, (2) NIR (808 nm, 1.5 W/cm^2^, 10 min), (c) CA-Fe NPs (400 μg/ml), and (4) CA-Fe NPs + NIR. After treatment, bacterial suspensions were serially diluted (10-fold), and 100-μl aliquots were plated on an LB agar plate. Bacterial viability was quantified by colony-forming unit (CFU) enumeration after overnight incubation.

### Live/dead bacterial staining

Bacteria were treated as described above and stained with SYTO9/propidium iodide (PI) for 30 min in the dark. Excess dye was removed by washing, and samples were visualized using laser scanning confocal microscopy. The live/dead ratio was quantified using GraphPad Prism 10.3.0.

### Protein leakage assay

Treated *E. coli* cells were centrifuged (20,000×*g*, 20 min). The protein concentration in the supernatant was determined using a bicinchoninic acid (BCA) protein assay kit (Thermo Scientific).

### Transcriptome sequencing

Total RNA was extracted from *S. aureus* (control vs. CA-Fe NPs + NIR groups, *n* = 4/group) and subjected to library construction, followed by RNA sequencing on the Illumina HiSeq platform. Raw data were filtered with fastp (v0.22.0) and aligned to the *S. aureus* genome using HISAT2 (v2.1.0). Gene expression was quantified via featureCounts (v1.6.0). Differentially expressed genes (DEGs) were identified using DESeq2 (|log_2_FoldChange| > 0.585, adjusted *P* < 0.05) and functionally annotated with the QuickGO database.

### Cell viability and ROS detection

RAW 264.7 cells (2 × 10^4^ cells/well) were treated with LPS and CA-Fe NPs (2.5 to 40 μg/ml) for 24 h. Cell viability was assessed using the Cell Counting Kit-8 assay. Human Kidney-2 (HK-2) cells were pretreated with CA-Fe NPs (15/30 μg/ml) followed by H_2_O_2_ (1.4 mM) stimulation. Intracellular ROS levels were detected using the 2′,7′-dichlorodihydrofluorescein diacetate (DCFH-DA) probe via fluorescence microscopy and flow cytometry (CytoFLEX; Beckman Coulter). Data were analyzed with ImageJ (v1.8.0) and FlowJo (v10.8.1).

### Apoptosis analysis

HK-2 cells were treated with H_2_O_2_ (1.4 mM) and CA-Fe NPs (10/20 μg/ml) for 24 h. Apoptosis was detected using an Annexin V–fluorescein isothiocyanate (FITC)/PI kit and analyzed via flow cytometry (CytoFLEX). Data were processed with FlowJo (v10.8.1).

### Western blot analysis

RAW 264.7 cell lysates were prepared using radioimmunoprecipitation assay buffer containing protease/phosphatase inhibitors. Proteins (quantified by BCA assay) were separated via 10% sodium dodecyl sulfate–polyacrylamide gel electrophoresis, transferred to 0.2-μm polyvinylidene difluoride membranes, and probed with specific primary and horseradish-peroxidase-conjugated secondary antibodies [22]. Protein bands were visualized using an enhanced chemiluminescence substrate.

### MRSA-infected wound model

Full-thickness dorsal wounds (8 mm) were created on BALB/c mice and inoculated with MRSA (40 μl, OD_600_ = 1.0). Mice were randomized into 4 treatment groups (*n* = 5) 24 h postinfection. Wound healing was photographed on days 0, 1, 3, 7, and 11. Wound tissues and organs were collected, fixed in 4% paraformaldehyde (PFA), and subjected to hematoxylin and eosin (H&E) and Masson’s trichrome staining for histopathological analysis.

### Hemolytic assay

Mouse blood cell suspensions (4%, v/v) were incubated with CA-Fe NPs (0 to 50 μg/ml) for 4 h. PBS and 1% Triton X-100 served as negative and positive controls, respectively. After centrifugation, the absorbance of the supernatant was measured at 542 nm. Hemolysis rate was calculated as follows: hemolysis (%) = (OD_(test)_ / OD_(positive)_) × 100%.

### Statistical analysis

All data are presented as the mean ± standard deviation from at least 3 independent experiments. Differences among groups were analyzed using one-way analysis of variance followed by Tukey post hoc test (GraphPad Prism 10.3.0). *P* <0.05 was considered statistically significant.

## Results and Discussion

### Preparation and characterization of CA-Fe NPs

**Fig. 2.**
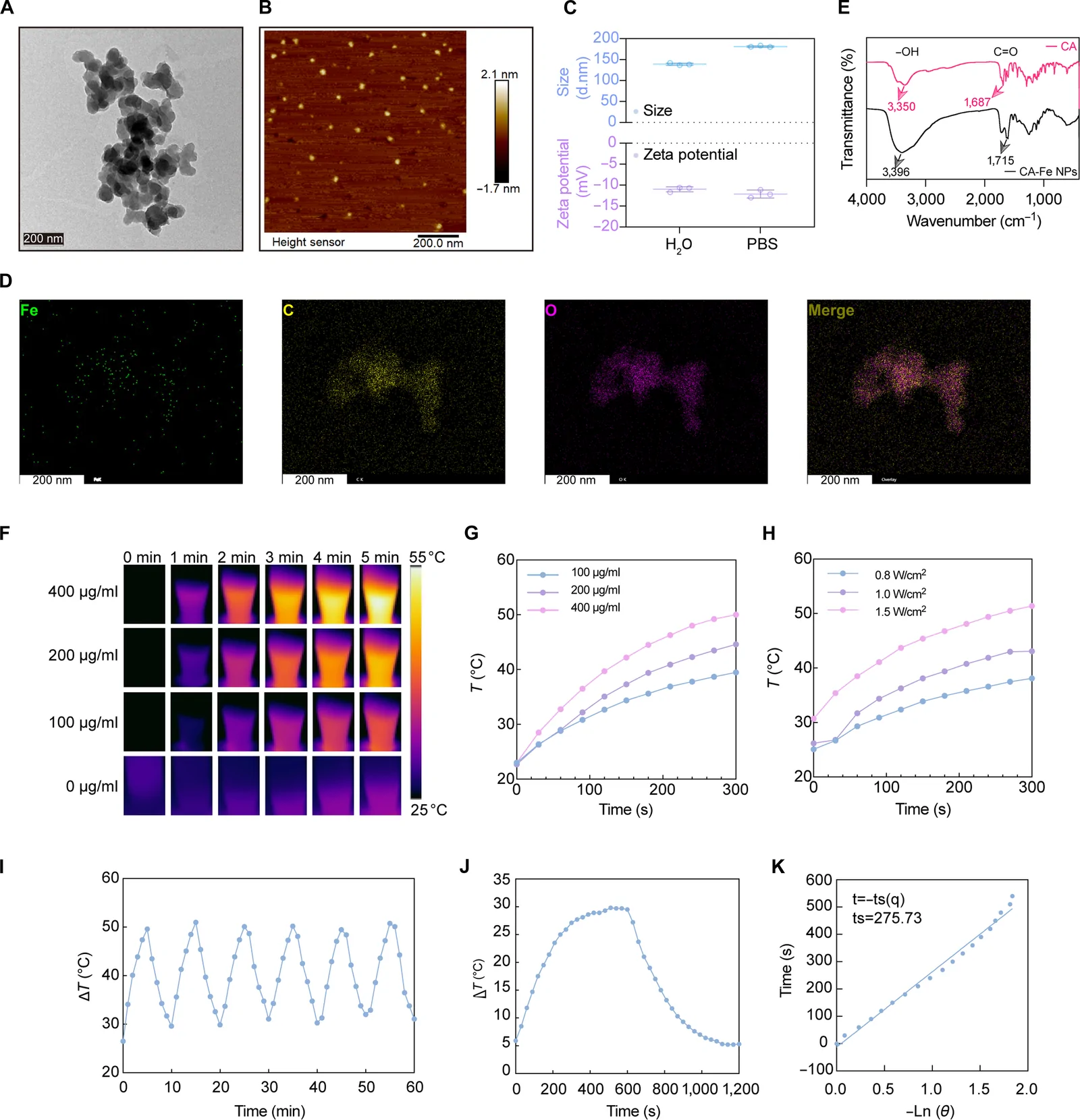
The characterization of CA-Fe NPs. (A) Transmission electron microscopy image of CA-Fe NPs. (B) Atomic force microscopy image of CA-Fe NPs. (C) Hydrodynamic size and zeta potential of CA-Fe NPs in H_2_O and phosphate-buffered saline (PBS). (D) Energy-dispersive x-ray spectroscopy elemental mapping images of CA-Fe NPs. (E) Fourier-transform infrared spectra of CA and CA-Fe NPs. (F) The infrared thermal images of CA-Fe NPs’ aqueous dispersions with different concentrations under an 808-nm laser (1.5 W/cm^2^) in vitro. (G) The temperature change of CA-Fe NPs’ aqueous dispersions with different concentrations irradiated by an 808-nm laser (1.5 W/cm^2^). (H) The temperature change of 400 μg/ml CA-Fe NPs’ aqueous dispersions with different power densities. (I) The photostability of CA-Fe NPs’ aqueous dispersions under an 808-nm laser irradiation for 6 heating/cooling cycles (1.5 W/cm^2^). (J) The photothermal performance of CA-Fe NPs’ aqueous dispersions under NIR irradiation (1.5 W/cm^2^). (K) Time constant for heat transfer.

According to previous reports, chlorogenic acid, a natural polyphenol-rich compound, demonstrated remarkable complexation capabilities with various metal ions, including ferrous and manganese ions [23]. Herein, CA-Fe NPs were fabricated via a nanoprecipitation-based self-assembly approach. Briefly, chlorogenic acid and FeCl_3_ were dissolved in absolute ethanol, sonicated until fully dissolved, and mixed, and then 5 ml of ultrapure water was added dropwise under stirring. Through metal-coordination chelation, chlorogenic acid and iron ions self-assembled into CA-Fe NPs with excellent aqueous dispersibility. Transmission electron microscopy analysis revealed nearly spherical morphology for CA-Fe NPs (Fig. 2A). The morphology and 3-dimensional structural characteristics of CA-Fe NPs were further verified by atomic force microscopy (Fig. 2B and Fig. S1). Atomic force microscopy characterization demonstrated that CA-Fe NPs presented uniform dispersion and monodisperse nanostructures, with a consistent nanoparticle height of approximately 2 nm. Dynamic light scattering and zeta-potential analyses were performed to evaluate the hydrodynamic size distribution and surface charge properties of CA-Fe NPs in ultrapure water and PBS (Fig. 2C). The average hydrodynamic size of CA-Fe NPs was 139.1 ± 2.79 nm in ultrapure water, which slightly increased to 181.33 ± 1.89 nm in PBS. Meanwhile, the zeta potentials of CA-Fe NPs were −11.0 ± 0.61 mV in ultrapure water and −12.1 ± 0.95 mV in PBS, displaying a slight negative shift in the physiological buffer environment. Notably, the variation trends of size and zeta potential of CA-Fe NPs were consistent with those of MPN NPs [24]. Energy-dispersive x-ray spectroscopy elemental mapping confirmed uniform distribution of C, O, and Fe within the nanoparticles (Fig. 2D). Fourier-transform infrared spectroscopy indicated key shifts compared to free CA: the –OH absorption peak shifted from 3,350 to 3,396 cm^−1^, while the carbonyl (C=O) absorption peak shifted from 1,687 to 1,715 cm^−1^ (Fig. 2E), preliminarily indicating coordination between chlorogenic acid and Fe^3+^. To further elucidate the coordination mechanism, x-ray photoelectron spectroscopy (XPS) analysis was performed. The XPS survey spectrum (Fig. S2) showed that pure chlorogenic acid exhibited only the characteristic peaks of C 1s (~285 eV) and O 1s (~532 eV), whereas CA-Fe NPs displayed additional peaks attributed to Fe 2p (~710 and ~724 eV), confirming the formation of stable chemical bonding rather than mere physical mixing between Fe and chlorogenic acid [25,26]. In the high-resolution Fe 2p spectrum, the binding energies of Fe 2p_3/2_ and Fe 2p_1/2_ were observed at 710.92 and 724.38 eV, respectively, consistent with the typical spin–orbit splitting pattern of Fe^3+^. In the high-resolution O 1s spectrum, the main O 1s peak of chlorogenic acid (532.84 eV) shifted negatively to 532.38 eV in CA-Fe NPs, with a shift of 0.46 eV [27]. This directly confirms that oxygen atoms from the hydroxyl/carboxyl groups of CA donate lone-pair electrons to Fe^3+^, forming Fe–O coordination bonds, which aligns with previously reported XPS characteristics of metal–phenol coordination [26]. Taken together, the XPS and earlier Fourier-transform infrared spectroscopy results thus unambiguously demonstrate the coordination between chlorogenic acid and Fe^3+^.

Previous literature established that NIR irradiation offers superior tissue penetration depth and reduced photodamage relative to ultraviolet light, enhancing its suitability for biological applications [28]. CA-Fe NPs synthesized under an optimized chlorogenic acid to iron molar ratio of 1:2 exhibited excellent aqueous dispersibility (polydispersity index: 0.16) and a uniform size distribution with a mean hydrodynamic diameter of 171 nm (Fig. S3). Under 808-nm NIR laser irradiation (1.5 W/cm^2^), CA-Fe NPs efficiently converted NIR light into thermal energy. Notably, the temperature of a 400 μg/ml dispersion increased from 23 to 50 °C within 5 min in a concentration-dependent manner, confirming excellent photothermal conversion capability (Fig. 2F and G). Further investigation under different laser power densities (0.8, 1.0, and 1.5 W/cm^2^) revealed temperature increases of 13, 16.9, and 20.7 °C, respectively, for the 400 μg/ml dispersion (Fig. 2H). Moreover, the photothermal response of CA-Fe NPs (400 μg/ml) remained stable across 6 consecutive heating/cooling cycles under NIR irradiation (1.5 W/cm^2^) (Fig. 2I), indicating exceptional stability as a photothermal agent for photothermal therapy. Finally, the photothermal conversion efficiency (*η*) was calculated based on the maximum temperature elevation (Fig. 2J) and the system’s heat transfer time constant (Fig. 2K), quantitatively substantiating the outstanding photothermal performance.

### Photothermal antibacterial performance of CA-Fe NPs in vitro

**Fig. 3.**
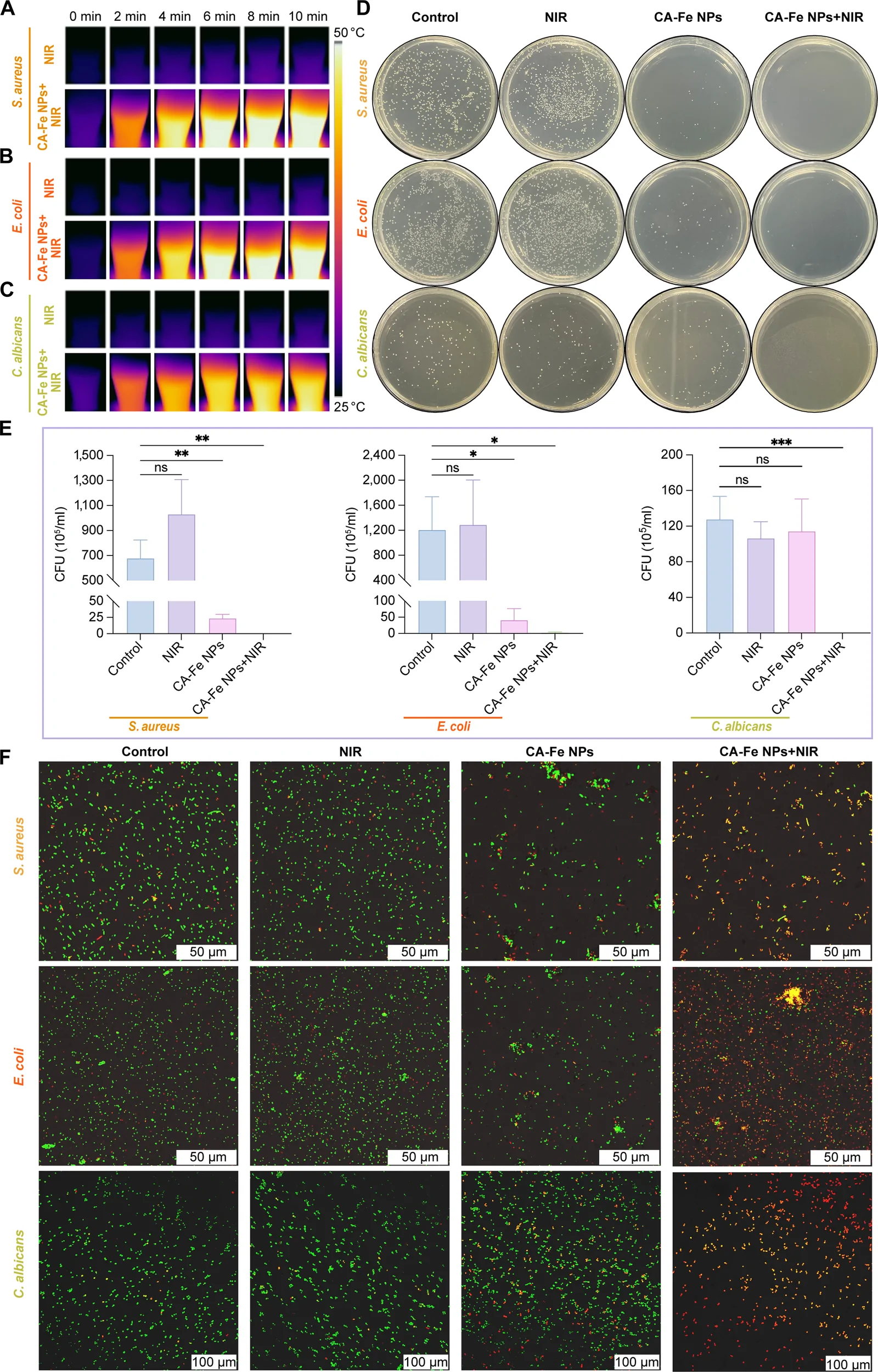
The photothermal antibacterial performance of CA-Fe NPs in vitro. (A to C) Infrared thermographic images of *S. aureus*, *Escherichia coli*, and *Candida albicans* suspensions under different treatment conditions: control (untreated), NIR alone, CA-Fe NPs, and CA-Fe NPs + NIR. (D) Representative images of microbial colonies. (E) Quantitative CFU of *S. aureus*, *E. coli*, and *C. albicans* under different treatment conditions: control (untreated), NIR alone, CA-Fe NPs, and CA-Fe NPs + NIR. (F) Viability staining (SYTO9/propidium iodide [PI]) of *S. aureus*, *E. coli*, and *C. albicans* under various conditions: control, NIR alone, CA-Fe NPs, and CA-Fe NPs + NIR. Statistical significance was analyzed by one-way analysis of variance (ANOVA). ns, not significant; *P < 0.0332; **P < 0.0021; ***P < 0.0002.

Based on the excellent photothermal properties of CA-Fe NPs, we conducted a series of experiments to investigate the photothermal antibacterial performance of CA-Fe NPs. As shown in Fig. 3A to C, the temperature of *S. aureus*, *E. coli*, and *C. albicans* solutions, treated with CA-Fe NPs and NIR irradiation (1.5 W/cm^2^, 10 min), increased by 28.9, 28.4, and 20.4 °C (Fig. S4), respectively. In contrast, solutions containing *S. aureus*, *E. coli*, and *C. albicans* exposed to NIR irradiation alone exhibited minimal temperature increases of only 3.5, 3.5, and 3.1 °C, respectively. Next, colony counting assays revealed sparse bacterial growth on LB agar plates for both *S. aureus* and *E. coli* treated with CA-Fe NPs (Fig. 3D and E), demonstrating strong antibacterial efficacy of the nanoparticles. In contrast, the efficacy against *C. albicans* was less pronounced, potentially due to the unique composition of its cell wall. Notably, when CA-Fe NPs were combined with NIR irradiation, a marked enhancement in antimicrobial activity was observed against all 3 pathogens. The average colony counts for *S. aureus*, *E. coli*, and *C. albicans* were reduced to 0, 2, and 0 CFU, respectively (Fig. 3E). Notably, NIR irradiation alone exhibited negligible antimicrobial activity against *S. aureus*, *E. coli*, and *C. albicans* when compared with untreated controls. To further assess the breadth of the antibacterial spectrum, we examined 2 additional critical Gram-negative ESKAPE pathogens, *P. aeruginosa* and *K. pneumoniae*, under consistent treatment conditions (CA-Fe NPs: 400 μg/ml; NIR: 808 nm, 1.5 W/cm^2^, 10 min). As shown in Fig. S5, the CA-Fe NPs + NIR treatment significantly reduced the viability of both pathogens. Collectively, these results confirm the broad-spectrum antibacterial capability of CA-Fe NPs. Additionally, bacterial viability was quantified using the LIVE/DEAD BacLight Bacterial Viability Kit (Thermo Fisher, Cat. No. L13152) to determine dead/live cell ratios (Fig. 3F and Figs. S6 to S8). Viable cells with intact membranes are stained green by SYTO9, while dead cells with compromised membranes are stained red by PI [29,30]. The CA-Fe NPs + NIR group exhibited the highest dead/live cell ratio, followed by the CA-Fe NPs group, whereas both the NIR-only and control groups showed minimal cell death (Fig. S9).

### Antibacterial mechanism of CA-Fe NPs

**Fig. 4.**
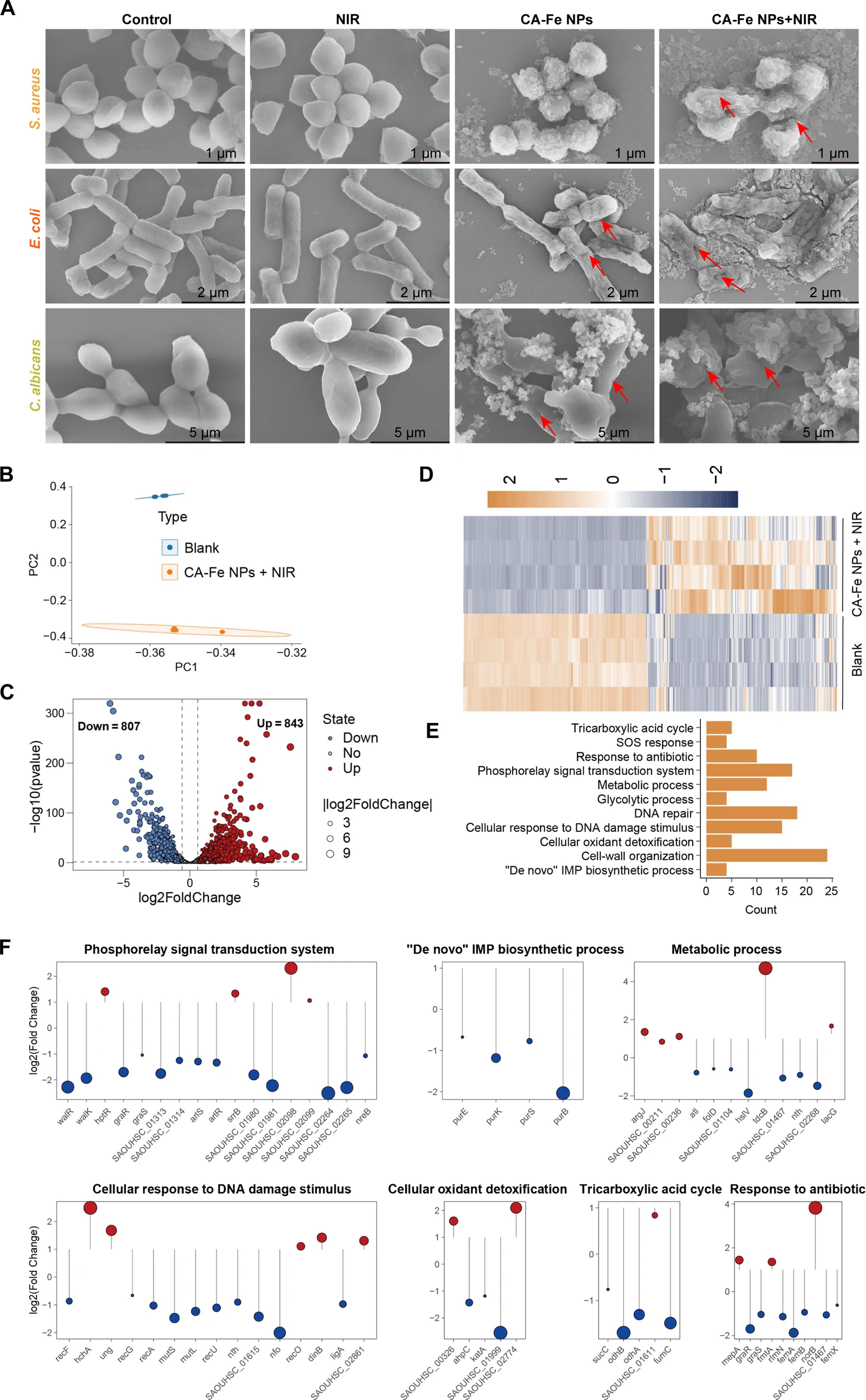
Antibacterial mechanism of CA-Fe NPs. (A) Scanning electron microscopy images of *S. aureus*, *E. coli*, and *C. albicans* under various treatments: untreated (control), NIR alone, CA-Fe NPs, and CA-Fe NPs + NIR. Red arrows indicate cell membrane collapse and even rupture. (B) Principal component (PC) analysis of RNA-sequence data of blank and CA-Fe NPs + NIR groups in *S. aureus*. (C) Volcano map of differentially up-regulated (red) and down-regulated (blue) genes. (D) Heatmap for the distribution of differentially expressed genes (DEGs) in different treatments. (E) Enrichment analysis of biological processes for DEGs. (F) Lollipop charts of the fold change of DEGs that are enriched in different pathways. Up-regulated genes are marked in red, while down-regulated genes are marked in blue. IMP, inosine monophosphate.

To investigate the potential antibacterial mechanism of CA-Fe NPs, we employed scanning electron microscopy to analyze the cellular morphological changes in *S. aureus*, *E. coli*, and *C. albicans* following different treatments. As shown in Fig. 4A, cells treated with CA-Fe NPs or CA-Fe NPs + NIR exhibited obvious membrane collapse and even rupture, accompanied by leakage of cellular contents. The damage was more severe in the CA-Fe NPs + NIR treatment group. In contrast, cells in the NIR-alone treatment group and the control group maintained intact structures and smooth surfaces. This indicates that CA-Fe NPs possess a strong ability to disrupt cell membranes, which is consistent with previous reports on the function of chlorogenic acid [31]. Next, we quantified protein leakage using a BCA assay. Results showed that the CA-Fe NPs + NIR group exhibited the highest protein leakage, followed by the CA-Fe NPs group. In contrast, both the NIR-only group and the control group showed negligible leakage (Fig. S10). These findings corroborate our scanning electron microscopy observations.

To further elucidate the antibacterial mechanism of CA-Fe NPs, we performed transcriptome analysis of *S. aureus* from the CA-Fe NPs + NIR treatment group and the blank (untreated) group. Principal component analysis revealed clear separation between the 4 biological replicates of the treatment and blank groups (Fig. 4B), indicating distinct transcriptomic profiles induced by the treatment. Differential expression analysis using DESeq2 (R package; |log2FoldChange| > 0.585, adjusted *P* value < 0.05) identified 1,650 DEGs (843 up-regulated and 807 down-regulated) between the 2 groups (Fig. 4C), with these DEGs exhibiting uniform distribution across the sample groups (Fig. 4D).

Functional enrichment analysis showed that these DEGs were primarily enriched in pathways including the tricarboxylic acid cycle, response to antibiotics, phosphorylation signal transduction systems, cell-wall organization, and other metabolic processes (Fig. 4E). Specifically, genes in the phosphorylation signaling system (*walK*/*walR*, *graS*/*graR*, and *arlS/arlR*) were down-regulated in the CA-Fe NPs + NIR group compared to the blank group (Fig. 4F). These regulatory genes are known to modulate critical processes, including cell-wall metabolism, multidrug resistance, virulence, biofilm formation, and oxidative-stress response. To independently verify the RNA-sequencing findings, we performed reverse transcription–quantitative polymerase chain reaction on selected DEGs. The results confirmed pronounced down-regulation of *walK*, *graR*, and *arlR* (by approximately 60%, 59%, and 80%, respectively) in the treated group (Fig. S11), strongly corroborating the transcriptomic profile. Consistent with these observations, genes associated with antibiotic response and oxidant detoxification were predominantly down-regulated. Moreover, the *purEKSB* genes, which play important roles in cellular purine metabolism, were all down-regulated in the de novo inosine monophosphate biosynthetic process. Furthermore, most genes involved in the cellular response to DNA damage stimuli were also down-regulated. Additionally, most genes implicated in the tricarboxylic acid cycle and metabolic processes were likewise down-regulated, which is in line with previous literature reports [32].

### Antioxidant and anti-inflammatory properties of CA-Fe NPs in vitro

**Fig. 5.**
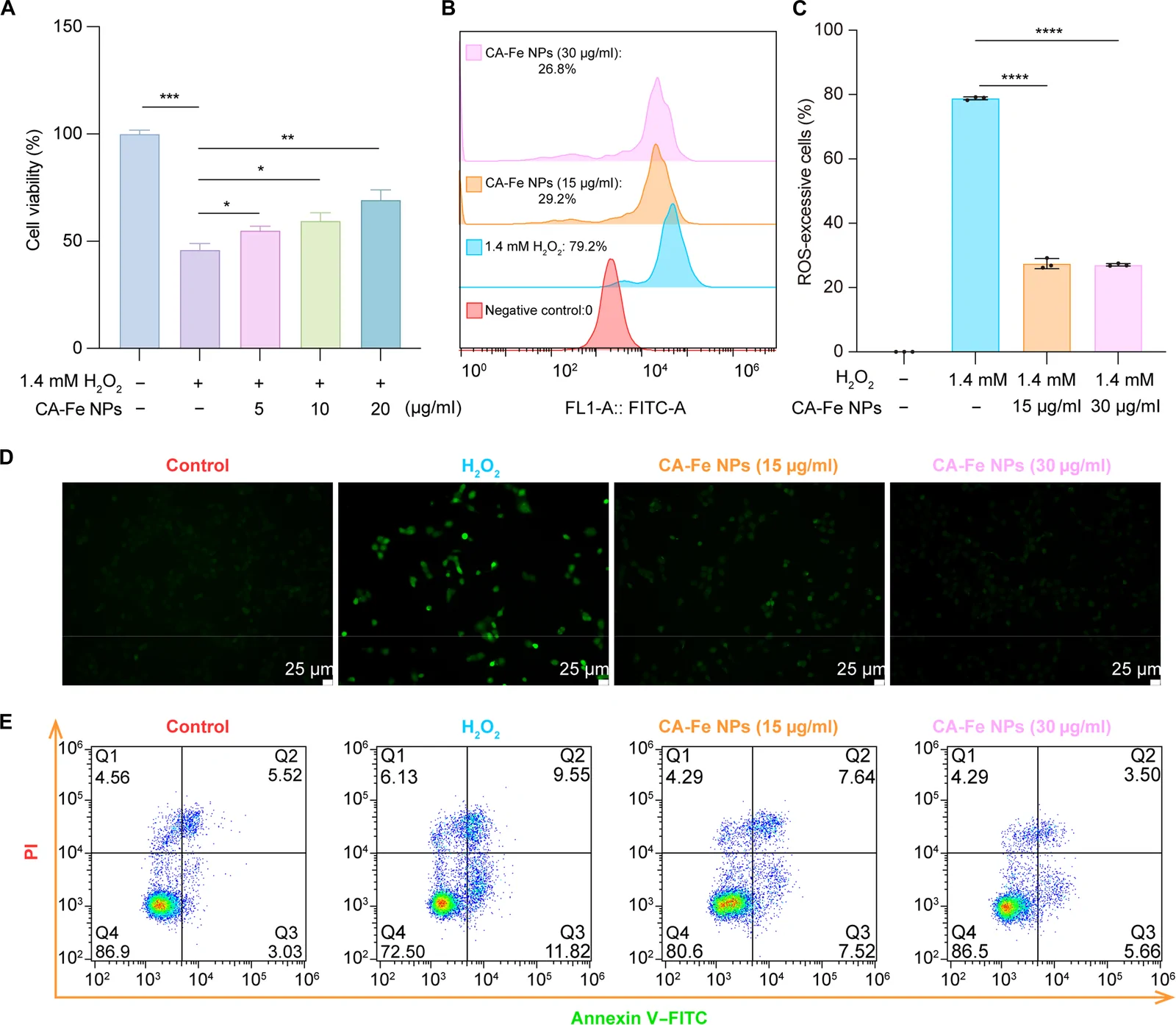
The anti-oxidation properties of CA-Fe NPs in vitro. (A) Viability of epithelial cells treated with H_2_O_2_ alone or H_2_O_2_ + CA-Fe NPs (0, 5, 10, and 20 μg/ml). (B) Intracellular ROS levels in epithelial cells under various treatments, analyzed by flow cytometry. (C) Proportion of ROS-positive epithelial cells under different treatments. (D) Fluorescence microscopy images of ROS staining in epithelial cells under various treatments. (E) Apoptosis in epithelial cells treated with H_2_O_2_ alone or H_2_O_2_ + CA-Fe NPs (10 or 30 μg/ml), analyzed by flow cytometry. FITC, fluorescein isothiocyanate. Statistical significance was analyzed by one-way ANOVA. *P < 0.0332; **P < 0.0021; ***P < 0.0002; ****P < 0.0001.

Previous studies have demonstrated that chlorogenic acid exhibits antioxidant properties in diverse cells [20,33]. To investigate the antioxidant potential of CA-Fe NPs, we first evaluated their SOD enzyme-like activity. At a concentration of 0.5 mg/ml, CA-Fe NPs exhibited 73.5% SOD activity (Fig. S12). Next, we assessed the protective effect of CA-Fe NPs on cell viability under H_2_O_2_-induced oxidative stress. As shown in Fig. 5A, CA-Fe NPs significantly protected cells from H_2_O_2_ toxicity in a concentration-dependent manner, enhancing cell viability. To further evaluate the antioxidant efficacy of CA-Fe NPs, intracellular ROS levels were measured using the DCFH-DA probe. Flow cytometry analysis revealed that CA-Fe NPs effectively reduced ROS levels compared to the H_2_O_2_ treatment group alone (Fig. 5B and C). Consistently, fluorescence microscopy imaging of epithelial cells showed a markedly weaker ROS fluorescence signal in the CA-Fe NPs-treated group than in the 1.4 mM H_2_O_2_-treated group (Fig. 5D). These combined results demonstrate that CA-Fe NPs possess remarkable antioxidant properties, protecting cells by scavenging ROS. Previous studies indicate that ROS accumulation can trigger programmed cell death, including apoptosis and necrosis [34,35]. To assess whether CA-Fe NPs protect cells from ROS-induced apoptosis, we performed Annexin V–FITC/PI staining followed by flow cytometry analysis. Phosphatidylserine, an apoptotic marker, translocated to the outer leaflet of the plasma membrane during apoptosis [36]. Annexin V–FITC specifically binds exposed phosphatidylserine in a calcium-dependent manner [37], while PI only stains cells with compromised membrane integrity, characteristic of late apoptosis and necrosis [38]. Flow cytometry revealed that CA-Fe NPs significantly reduced the proportion of apoptotic cells compared to the 1.4 mM H_2_O_2_ group (Fig. 4E and Fig. S13). Notably, the apoptosis rate in the 30 μg/ml CA-Fe NPs group decreased to the level of the control group (Fig. S13). These results demonstrate that CA-Fe NPs effectively rescue epithelial cells from ROS-induced death by scavenging ROS and suppressing apoptosis.

**Fig. 6.**
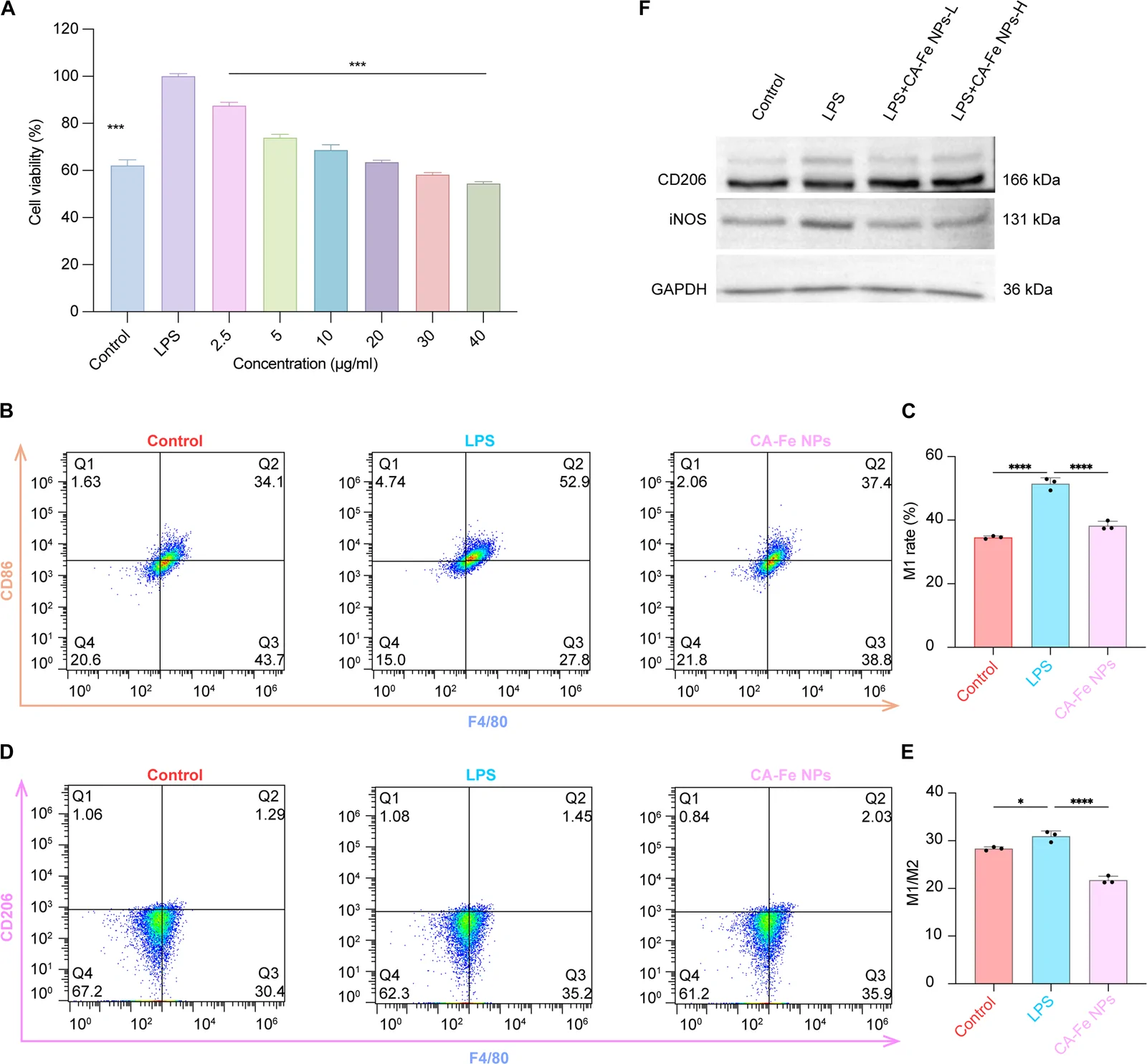
The anti-inflammatory properties of CA-Fe NPs in vitro. (A) The proportion of macrophage recruitment under lipopolysaccharide (LPS) or LPS + CA-Fe NPs treatment. (B) Flow cytometric analysis of M1 macrophages under various treatments. (C) Ratio of M1 macrophages. (D) Flow cytometric analysis of M2 macrophages under various treatments. (E) Ratio of M1/M2 macrophages. (F) Expression levels of CD206 (M2 marker) and inducible nitric oxide synthase (iNOS, M1 marker) were detected by Western blotting. Glyceraldehyde-3-phosphate dehydrogenase (GAPDH) was used as the loading control. Statistical significance was analyzed by one-way ANOVA. *P < 0.0332; ***P < 0.0002; ****P < 0.0001.

Chlorogenic acid, attributed to its polyphenolic structure, exhibits not only antioxidant but also anti-inflammatory properties [39]. To investigate the anti-inflammatory potential of CA-Fe NPs, we conducted several experiments. As shown in Fig. 6A, CA-Fe NPs significantly and concentration-dependently reduced the LPS-induced recruitment of macrophages. We further assessed the impact of CA-Fe NPs on macrophage polarization using flow cytometry. The results demonstrate that CA-Fe NPs suppressed polarization toward the pro-inflammatory M1 phenotype while promoting polarization toward the anti-inflammatory M2 phenotype (Fig. 6B to E). M1 macrophages are typically pro-inflammatory, playing key roles in early inflammation, anti-infection, anti-tumor responses, and clearance of apoptotic/necrotic debris [40]. In contrast, M2 macrophages mediate anti-inflammatory responses and Th2 immunity, crucial for inflammation resolution and tissue repair [41]. Consistent with the flow cytometry data, Western blot analysis yielded similar results (Fig. 6F and Fig. S14). Collectively, these findings indicate that CA-Fe NPs effectively mitigate oxidative stress not only by reducing intracellular ROS levels but also by modulating macrophage polarization—specifically, inhibiting M1 polarization and promoting M2 polarization.

### CA-Fe NPs for in vivo treatment of MRSA-infected wound healing

**Fig. 7.**
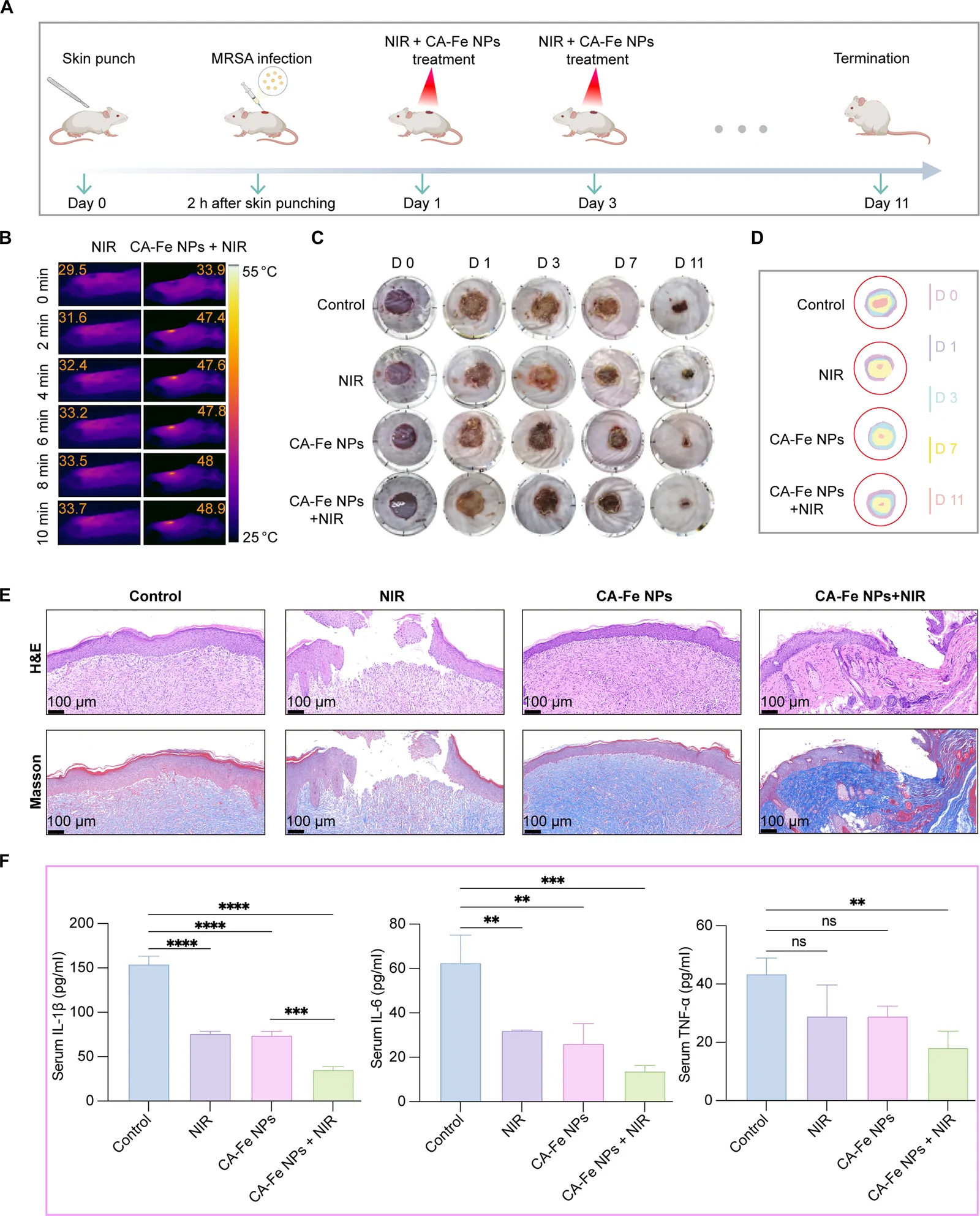
In vivo therapeutic efficacy of CA-Fe NPs on MRSA-infected wounds. (A) Schematic of *S. aureus*-infected wound model establishment and treatment protocol. (B) Infrared thermal images of MRSA-infected wounds with/without CA-Fe NPs under NIR irradiation. (C) Wound morphology in MRSA-infected mice after indicated treatments on days 0, 1, 3, 7, and 11. (D) Quantification of wound area. (E) Representative hematoxylin and eosin (H&E) and Masson’s trichrome-stained sections of wound tissues across groups. (F) Serum tumor necrosis factor alpha (TNF-α), interleukin-1 beta (IL-1β), and interleukin-6 (IL-6) levels were measured by enzyme-linked immunosorbent assay in control, NIR, CA-Fe NPs, and CA-Fe NPs + NIR groups. Statistical significance was analyzed by one-way ANOVA. ns, not significant; **P < 0.0021; ***P < 0.0002; ****P < 0.0001.

Based on the prior in vitro experimental results demonstrating remarkable antibacterial, antioxidant, and anti-inflammatory efficacy of CA-Fe NPs, we established a mouse model of MRSA-infected wounds to evaluate their effect on wound healing (Fig. 7A). MRSA is a prevalent pathogen causing skin infections [42]. One day postinfection with MRSA, mice underwent the following treatments: NIR alone, CA-Fe NPs alone, and CA-Fe NPs + NIR. We found that CA-Fe NPs also exhibited excellent photothermal performance in vivo. The temperature of wounds treated with CA-Fe NPs + NIR increased by 15 °C, while the temperature in wounds treated only with NIR increased by 4.2 °C (Fig. 7B). Mice receiving the combined CA-Fe NPs + NIR treatment showed a considerably higher wound-healing rate compared to those treated with CA-Fe NPs alone, NIR alone, or the control group (Fig. 7C and D). Quantitative analysis of CFU in wound tissues on day 4 posttreatment directly demonstrated that the bacterial load in the CA-Fe NPs + NIR group was significantly lower than in the CA-Fe NPs alone group (*P* < 0.05, Fig. S15). This confirms that under complex in vivo conditions, NIR-induced photothermal effects substantially enhance the local antibacterial efficacy of CA-Fe NPs, consistent with the synergistic bactericidal mechanism observed in vitro. These results indicate that CA-Fe NPs effectively enhance wound healing. Furthermore, we assessed wound healing histologically across different groups using H&E staining and Masson’s trichrome staining [43]. Histological analysis revealed that granulation tissue formation and collagen deposition were markedly higher in both the CA-Fe NPs group and the CA-Fe NPs + NIR group compared to the NIR group and the control group (Fig. 7E). Granulation tissue formation [44] and collagen deposition [45] are critical indicators of wound recovery [46]. Finally, the systemic inflammatory response was evaluated by measuring serum inflammatory factors. Levels of interleukin-1 beta, interleukin-6, and tumor necrosis factor alpha in mouse serum were significantly lower in the CA-Fe NPs + NIR group than in the control group (Fig. 7F). Comparison of the results among different treatment groups reveals that the outstanding therapeutic efficacy of CA-Fe NPs can be ascribed to the synergistic effect between their photothermal antibacterial activity and inherent bioactive characteristics. Compared with the control group, animals treated with CA-Fe NPs alone showed accelerated wound closure, improved tissue formation, and alleviated systemic inflammation, confirming the crucial roles of their intrinsic anti-inflammatory, antioxidant, and pro-repair properties in the healing process. Notably, the CA-Fe NPs + NIR group exhibited the most rapid and complete wound repair. We propose a sequential cooperative mechanism: In the early stage, the rapid photothermal bactericidal effect efficiently reduces bacterial load and establishes a low-infection microenvironment. In the mid-to-late healing phase, the sustained antioxidant and immunomodulatory effects derived from released chlorogenic acid play a dominant role in promoting tissue maturation and remodeling. Therefore, photothermal therapy eliminates the infectious barrier to facilitate the function of bioactive components, while the inherent bioactivities guarantee subsequent effective tissue restoration. In conclusion, CA-Fe NPs demonstrate remarkable therapeutic efficacy in treating MRSA-infected wounds, with the combined CA-Fe NPs + NIR treatment achieving the best outcome through the synergistic effect of photothermal bactericidal activity and inherent bioactive properties.

We evaluated the biocompatibility of CA-Fe NPs using epithelial cells. Even at a high concentration of 100 μg/ml, cell viability showed no obvious reduction (Fig. S16). Moreover, hemolysis assays demonstrated excellent blood compatibility of CA-Fe NPs (Fig. S17). H&E staining analysis of heart, liver, spleen, lung, and kidney tissues from mice in all 4 groups on day 11 revealed no apparent morphological abnormalities (Fig. S18). These findings demonstrate that CA-Fe NPs are safe, highly efficacious, and low-toxicity antibacterial agents.

## Conclusion

This study developed CA-Fe NPs, a natural polyphenol–metal formulation integrating antibacterial, antioxidative, and anti-inflammatory properties for treating wound infections. Chlorogenic acid—an active compound derived from traditional Chinese medicine—self-assembles with iron ions to form uniform, stable nanoparticles. In vitro, CA-Fe NPs demonstrated potent eradication of *S. aureus* (Gram-positive), *E. coli* (Gram-negative), *C. albicans* (fungi), *K. pneumoniae* (Gram-negative), and *P. aeruginosa* (Gram-negative) via photothermal sterilization. Additionally, CA-Fe NPs markedly reduced ROS levels in epithelial cells, thereby attenuating apoptosis. Furthermore, they effectively suppressed LPS-induced polarization of macrophages toward the M1 phenotype. In vivo, using an MRSA-infected mouse wound model, CA-Fe NPs alleviated inflammation and accelerated wound healing while exhibiting excellent biocompatibility and low cytotoxicity. Collectively, CA-Fe NPs represent a promising photothermal therapeutic platform for the clinical management of wound infections.

## Data Availability

All data supporting the findings of this study are contained within the paper and its supplementary information files.
