# Supplementary material for: Multifunctional Metal Polyphenol Nanoparticles Based on Photothermal Therapy for Bacterial Eradication and Wound Healing
Source: Biomater Res. 2026 Sep 1;30:0387. doi: 10.34133/bmr.0387 (PMC13530372; doi:10.34133/bmr.0387)
Supplement: Supplementary file 1 [file bmr.0387.f1.docx]

**Supplementary information**

Multifunctional Metal Polyphenol Nanoparticles Based on Photothermal Therapy for Bacterial Eradication and Wound Healing

Haitao Yuan ^1,3#^, Mengyun Hou ^2,3#^, Min Zou ^2#^, Shiyan He ^3^, Yunmeng Bai ^3^, Wenzhe Chen ^1^, Junhui Chen ^3^, Xiaoxian Wang ^3^, Jingbo Ma ^3^, Jinyue He ^2^, Centing Wang ^1^, Xinmiao Liu ^3^, Lihong Jiang ^6*^, Li Song ^3*^, Wei Xiao ^1*^, Jigang Wang ^1,2,3,4,5*^

^1^ Center for Drug Research and Development, Guangdong Provincial Key Laboratory for Research and Evaluation of Pharmaceutical Preparations, Guangdong Pharmaceutical University, Guangzhou 510006, P.R. China.

^2^ Department of Clinical Laboratory, Guangdong Provincial Clinical Research Center for Geriatrics, Shenzhen Clinical Research Center for Geriatrics, Shenzhen People’s Hospital (The Second Clinical Medical College, Jinan University; The First Affiliated Hospital, Southern University of Science and Technology), Shenzhen 518020, P.R. China.

^3^ Guangdong Provincial Key Laboratory of New Drug Screening, School of Pharmaceutical Sciences, Southern Medical University, Guangzhou 510515, P.R. China.

^4^ Center for Translational Research in Clinical Medicine, Medical School, Kunming University of Science and Technology, Kunming, P.R. China.

^5^ State Key Laboratory for Quality Ensurance and Sustainable Use of Dao-di Herbs, Artemisinin Research Center, and Institute of Chinese Materia Medica, China Academy of Chinese Medical Sciences, Beijing 100700, P.R. China.

^6^ Shandong Academy of Chinese Medicine, Jinan 250000, P.R. China.

^#^ Haitao Yuan, Mengyun Hou and Min Zou contributed equally to this work.

^*^Correspondence: 158987215@qq.com (Li Song), xw2015103@kust.edu.cn (Lihong Jiang), xw7688@smu.edu.cn (Wei Xiao), jgwang@icmm.ac.cn (Jigang Wang)

Fig. S1. Three-dimensional atomic force microscopy (AFM) height image of CA-Fe NPs. The scan area is 1.0 μm × 1.0 μm, with a vertical height range from -1.7 nm to 2.1 nm.

Fig. S2. XPS analysis confirming the coordination between chlorogenic acid (CA) and Fe³⁺. (a) XPS survey spectra of pure CA and CA-Fe NPs. (b) High-resolution Fe 2p spectrum of CA-Fe NPs. (c) High-resolution C 1s spectra of CA and CA-Fe NPs. (d) High-resolution O 1s spectra of CA and CA-Fe NPs.

Fig. S3. Structural and photothermal properties of chlorogenic acid‑iron (CA‑Fe) nanoparticles prepared at different CA: Fe molar ratios (1:1, 1:2, 1:3). (a) Hydrodynamic size distribution determined by dynamic light scattering (DLS); (b) Quantitative Z‑average hydrodynamic diameter; (c) Corresponding polydispersity index (PDI); (d) Photothermal heating profiles under 808 nm NIR laser irradiation (1.5 W/cm²) over 5 min.

Fig. S4. Temperature changes of bacterial/fungal solutions treated with NIR or CA-Fe NPs + NIR. (a) The temperature change of the *S. aureus* solution treated with NIR or CA-Fe NPs + NIR. (b) The temperature change of the *E. coli* solution treated with NIR or CA-Fe NPs + NIR. (c) The temperature change of *the C. albicans* solution treated with NIR or CA-Fe NPs + NIR.

Fig. S5. Antibacterial activity of CA-Fe NPs alone or in combination with NIR irradiation against *P. aeruginosa* and *K. pneumoniae*. (a, b) Colony formation assays of *P. aeruginosa* (a) and *K. pneumoniae* (b) treated under different conditions: Control (untreated), NIR irradiation alone, CA-Fe NPs alone, and CA-Fe NPs with NIR irradiation. (c, d) Quantitative analysis of CFU corresponding to (a) and (b), respectively.

Fig. S6. Confocal laser scanning microscopic (CLSM) images of *S. aureus* stained with the LIVE /DEAD BacLight staining kits after various treatments.

Fig. S7. Confocal laser scanning microscopic (CLSM) images of *E. coli* stained with the LIVE /DEAD BacLight staining kits after various treatments.

Fig. S8. Confocal laser scanning microscopic (CLSM) images of *C. albicans* stained with the LIVE /DEAD BacLight staining kits after various treatments.

Fig. S9. The ratio of dead/live bacteria and fungi treated with different treatments. (a) The ratio of dead/live of *S. aureus* under NIR, CA-Fe NPs, CA-Fe NPs + NIR treatments. (b) The ratio of dead/live *E. coli* under NIR, CA-Fe NPs, CA-Fe NPs + NIR treatments. (c) The ratio of dead/live of *C. albicans* under NIR, CA-Fe NPs, CA-Fe NPs + NIR treatments.

Fig. S10. Protein leakage of *E. coli* under various treatments.

Fig. S11. RT‑qPCR validation of key differentially expressed genes in MRSA after CA‑Fe NPs + NIR treatment.

Fig. S12. The SOD-like activity of CA/CA-Fe NPs.

Fig. S13. The proportion of apoptotic **epithelial cells** under H_2_O_2_ or H_2_O_2_ + CA-Fe NPs (15 μg/mL or 30 μg/mL) treatments.

Fig. S14. CA-Fe NPs modulate the expression of M2 and M1 macrophage markers in LPS-induced inflammation (a) The CD206 relative expression level of RAW 264.7 cells under different treatments. (b) The iNOS relative expression level of RAW 264.7 cells under different treatments

Fig. S15. CFU counts in wound tissues on day 4 post-treatment.

Fig. S16. Cell viability assessment of **epithelial cells** treated with CA-Fe NPs.

Fig. S17. Hemolysis test of CA-Fe NPs.

Fig. S18. Histological analysis of heart, liver, spleen, lung, and kidney by H&E staining.
